# Influence of the Hydrolyzable Tannin Structure on the Characteristics of Insoluble Hydrolyzable Tannin–Protein Complexes

**DOI:** 10.1021/acs.jafc.2c01765

**Published:** 2022-06-16

**Authors:** Marica T. Engström, Valtteri Virtanen, Juha-Pekka Salminen

**Affiliations:** Natural Chemistry Research Group, Department of Chemistry, University of Turku, FI-20014 Turku, Finland

**Keywords:** bovine serum albumin, complex composition, cooperative binding, hydrolyzable tannin, insoluble complex, protein precipitation, stoichiometry

## Abstract

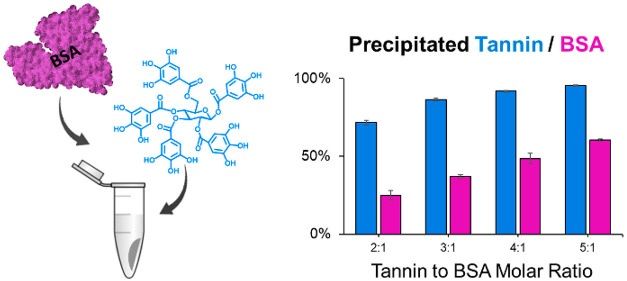

Precipitation of bovine serum albumin (BSA) by 21 hydrolyzable tannins (HTs) and the characteristics of the insoluble complexes were studied stoichiometrically by ultra-performance liquid chromatography. With regard to HT monomers, the protein precipitation and the characteristic of the formed precipitates were unique for each studied HT and depended upon the functional groups present in the structures. The monomeric units comprising the oligomers formed the functional units important for the protein precipitation capacity, and small structural differences among the monomer units were less important than the overall oligomer size and flexibility. In addition, the greater tendency of certain HTs to form insoluble complexes when mixed with BSA was partially linked to the higher self-association and consequent stronger cooperative binding of these HTs with BSA.

## Introduction

Tannins, which are classified into two major groups, hydrolyzable tannins (HTs) and proanthocyanidins, are a complex class of polyphenols and are found in a wide range of plant species.^1,2^ The tendency to interact and form insoluble precipitates with proteins and other biological macromolecules in aqueous solutions is the significant characteristic that distinguishes tannins from other natural polyphenols.^3,4^ In addition to the importance in defense mechanisms of plants,^5−7^ tannin–protein interactions have great importance in the numerous bioactivities and their possible applications for human and animal nutrition and health. To better understand the interplay of tannins and proteins in the complex formation, it has been important to determine the features affecting the complex formation.^8−10^

Since the pioneering studies of Haslam,^11^ the importance of the tannin structure in tannin–protein interactions has been explored and reviewed in numerous studies.^4,12−25^ In our previous study, we showed that the ability of HTs to form insoluble complexes with bovine serum albumin (BSA), measured with a turbidimetric method, could be predicted from the HT structure with high accuracy.^24^ The main features affecting the protein precipitation capacity (PPC) of monomeric HTs included the number of galloyl and other galloyl-derived functional groups, degree of oxidative coupling between the galloyls, positional isomerism, cyclic versus acyclic glucose core, and molecular weight (MW) that reflected the number of the phenolic functional units in the HTs. With regard to the oligomeric ellagitannins (ETs) studied, their PPC depended mostly upon their size and overall flexibility. The modest number of variable phenolic functional groups bound to the core polyol, in the oligomer versus oligomer comparisons, did not allow us to determine the specific effects of these individual functional groups on the PPC of oligomeric ETs. However, it appeared that, with the oligomers, the monomers now formed the functional units important for the PPC, and small differences between the monomers were less important than the overall oligomer size and also flexibility. Our results confirmed the previous findings from various papers and in addition complemented the previous works on how the PPC of HTs is reflected in their biosynthetic pathway.^14,16,17^

In addition to explaining the PPC of HTs by their structural features, the characteristics of the precipitates could yield interesting information on how precipitation differs from HT to HT and if complex compositions are directly proportional to the PPC of different HTs. For a more detailed study of the complex compositions in the precipitates formed after mixing HT and protein, the residual tannin or protein in the supernatant,^26−28^ in the precipitate,^16,29−32^ or in both^33−35^ can be analyzed. High-performance liquid chromatography (HPLC) can be effectively used to quantitatively study the characteristics of the formed precipitates and has been used to study HT–protein complexes to established details on the interaction between galloyl glucoses and serum albumin.^16,30,33−35^ In the present study, we selected 21 HTs based on their PPC and examined, in more detail using ultra-high-performance liquid chromatography coupled with diode array detection (UHPLC–DAD), how the HT structure affects the characteristics of insoluble HT–protein complexes.

## Materials and Methods

### Chemicals

Technical-grade acetone used for extraction was purchased from VWR (Haasrode, Belgium). Analytical-grade acetone and methanol used in the Sephadex LH-20 fractionation as well as HPLC-grade methanol and acetonitrile used in the preparative and semi-preparative purification were from VWR International (Fontenay-Sous-Bois, France). Formic acid and liquid chromatography–mass spectrometry (LC–MS) Chromasolv acetonitrile used in UHPLC–DAD and UHPLC–DAD–electrospray ionization (ESI)–triple quadrupole (QqQ) analyses were obtained from Sigma-Aldrich (Seelze, Germany). All water used was purified with a Millipore Synergy water purification system from Merck KGaA (Darmstadt, Germany). Sephadex LH-20 material was purchased from GE Healthcare (Uppsala, Sweden). BSA (purified by heat-shock fractionation, pH 7, purity of ≥96%; lyophilized powder, 66 kDa) was purchased from Sigma-Aldrich (St. Louis, MO, U.S.A.).

### Plant Materials

The plant materials used for the isolation of the studied HTs were the same as in the study by Engström et al.^24^ and were collected during summers of 2011–2017 from Southwestern Finland. The plant materials were willowherb (*Chamaenerion angustifolium*) flowers, silverweed (*Potentilla anserina*) leaves, herb Bennet (*Geum urbanum*) leaves, English oak (*Quercus robur*) acorns, purple loosestrife (*Lythrum salicaria*) leaves and flowers, meadowsweet (*Filipendula ulmaria*) flowers, raspberry (*Rubus idaeus*) leaves, and wood cranesbill (*Geranium sylvaticum*) leaves. Black myrobalan (*Terminalia chebula*) powder was purchased from Banyan Botanicals (Albuquerque, NM, U.S.A.). Sea buckthorn (*Hippophae rhamnoides*) leaves were the same as used in the study by Moilanen et al.,^36^ and white birch (*Betula pubescens*) leaf material was the same as in the study by Salminen et al.^37^

### HT Isolation

**Figure 1:**
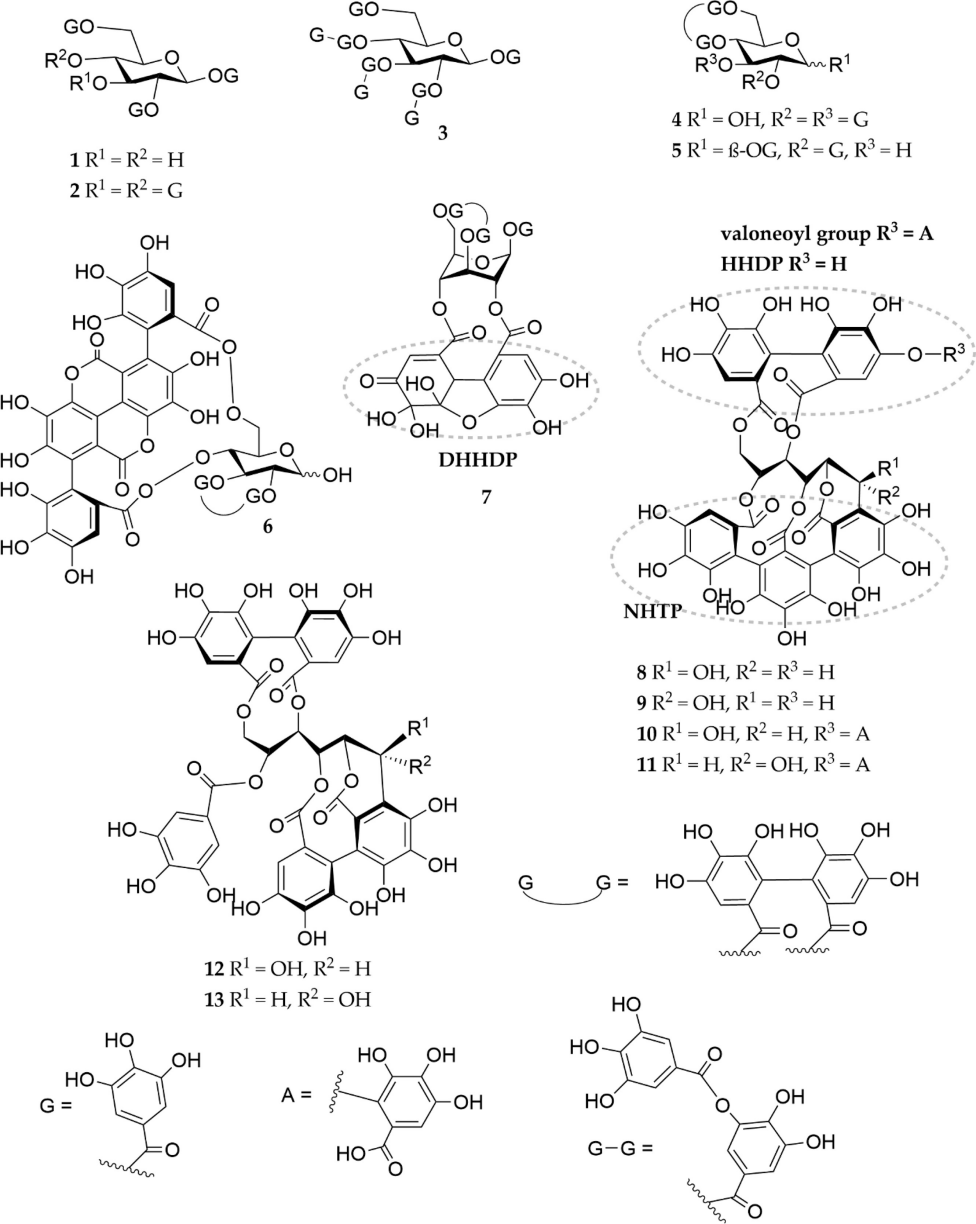
Structures of the monomeric HTs studied: 1,2,6-tri-*O*-galloyl-β-d-glucose (**1**), 1,2,3,4,6-penta-*O*-galloyl-β-d-glucose (**2**), octagalloylglucose (**3**), tellimagrandin I (**4**), 1,2-di-*O*-galloyl-4,6-HHDP-β-d-glucose (**5**), punicalagin (**6**), geraniin (**7**), vescalagin (**8**), castalagin (**9**), vescavaloninic acid (**10**), castavaloninic acid (**11**), stachyurin (**12**), and casuarinin (**13**). HHDP, hexahydroxydiphenoyl; DHHDP, dehydrohexahydroxydiphenoyl; and NHTP, nonahydroxytriphenoyl.

**Figure 2:**
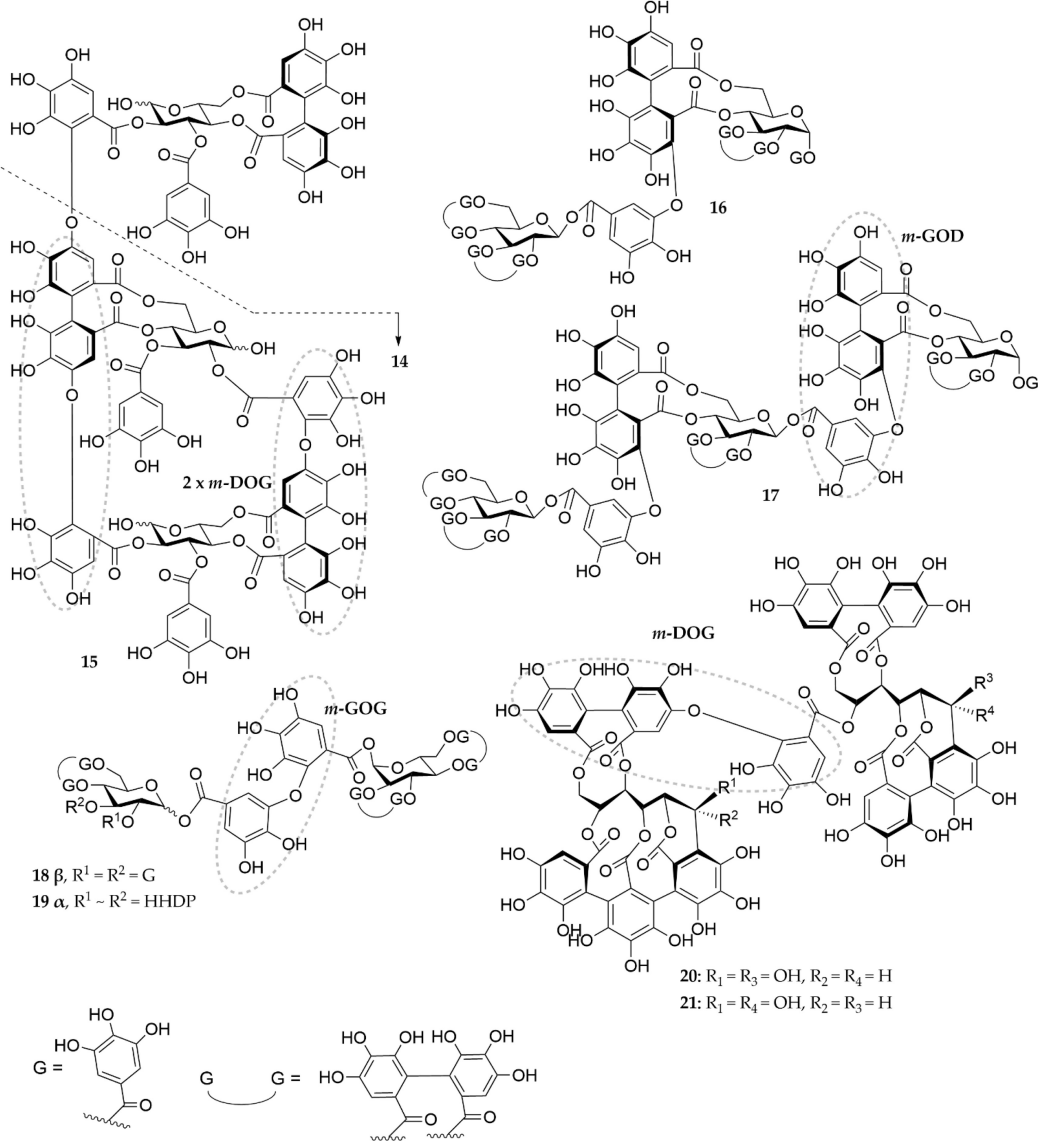
Structures of the oligomeric HTs studied and the different types of linkages between the monomers oenothein B (**14**), oenothein A (**15**), sanguiin H-6 (**16**), lambertianin C (**17**), gemin A (**18**), agrimoniin (**19**), salicarinin A (**20**), and salicarinin B (**21**). *m*-DOG, valoneoyl group; *m*-GOG, dehydrodigalloyl group; *m*-GOD, sanguisorboyl group; and 2× *m*-DOG, macrocyclic structure.

The extraction of the plant materials and the isolation and identification of the HTs (Figures 1 and 2) used in this paper were performed as described in the studies by Baert et al.^38^ and Engström et al.^24^ Briefly, the plant materials were collected into 1 L glass bottles filled with acetone and macerated at 4 °C. Maceration was repeated with additional batches of acetone/water (4:1, v/v); the different extraction batches were combined; acetone was evaporated; and the remaining aqueous solution was filtered and lyophilized. For the first crude fractionation, the lyophilized extracts were dissolved in water, mixed to a slurry of Sephadex LH-20 (in water) material, and sequentially eluted and vacuum-filtered with water, methanol/water (1:1, v/v), methanol, acetone/water (4:1, v/v), and acetone in a Büchner funnel (⌀ = 240 mm) with a filter paper. Subsequent fractionation was performed in a glass column loaded with Sephadex LH-20 gel, which was dissolved and stabilized in water. The eluents used in fractionation were ultrapure water, aqueous methanol, and aqueous acetone, and the elution profile depended upon the HT to be isolated. The obtained fractions were analyzed by UHPLC–DAD–ESI–MS, concentrated to the water phase, and lyophilized. Final purifications for selected Sephadex fractions were performed with preparative and semi-preparative liquid chromatography. All purification steps and purities of final products were monitored with UHPLC–DAD–ESI–MS. The structures of the individual HTs are presented in Figures 1 and 2. Information on the original plant material, the purity by UHPLC–DAD at 280 nm, the ESI–MS identification, and the original structural identification papers of the studied 21 HTs are presented in the S1 Appendix of the Supporting Information.

### HT Selection

A total of 21 HTs (Figures 1 and 2) were selected for the study based on the PPC results in the study by Engström et al.^24^ Only HTs with the ability to precipitate proteins at a HT/BSA ratio lower than 5:1 were selected because the aim of this study was to investigate the complex compositions of the formed precipitates. Purities of the selected HTs varied between 89 and 100%, as measured by UHPLC–DAD. The monomers were represented by 13 HTs: 7 with central glucose in pyranose form (**1**–**7**) and 6 with open chain form *C*-glycosidic ETs (**8**–**13**). These included two simple galloyl glucoses (**1** and **2**) and one gallotannin (**3**). Simple hexahydroxydiphenoyl (HHDP) esters were represented by three ellagitannins (ETs, **4**–**6**), each carrying the characteristic feature of ETs, the HHDP moiety, and in addition, compound **6** containing a gallagyl group. The oxidized form of HHDP, dehydrohexahydroxydiphenoyl group (DHHDP), was present in compound **7**.

The eight oligomeric ETs were constructed of either simple HHDP esters or *C*-glycosidic monomers and represented five different oligomer types. Compounds **14** and **15** were macrocyclic oligomers: compound **14** with two *m*-DOG linkages and compound **15** with one *m*-DOG and one *m*,*m*′-D(OG)_2_ linkage. Sanguisorboyl groups (*m*-GOD) were found in compounds **16** and **17**, and dehydrodigalloyl groups (*m*-GOG) were found in compounds **18** and **19**. Compounds **20** and **21** were selected to represent *C*-glycosidic oligomers containing an intermolecular valoneoyl group (*m*-DOG).

### Protein Precipitation Capacity and Complex Composition

The turbidimetric plate reader PPC measurements of the studied HTs were performed as in the study by Engström et al.^24^ Initially, the PPCs were measured at HT/protein ratios of 2:1, 3:1, 4:1, and 5:1. The BSA concentration was constant, 100 μM in the reaction mixture, and the pH of the reaction solution was 5. If the PP_50_ value (HT concentration at which 50% of the protein present has precipitated) was not achieved within these molar ratios, HTs with high PPC were further tested at 1:1 and 1:2 molar ratios, and HTs with low PPC were further tested at 6:1, 7.5:1, 9:1, and 10:1. For each HT/BSA ratio studied, three replicate reactions were performed, and after the incubation, the whole sample in each well was transferred into microcentrifuge tubes and centrifuged 10 min at 16000*g* (Centrifuge 5402, Eppendorf AG, Hamburg, Germany). After centrifugation, subsamples were carefully taken from the supernatant, filtered with a syringe filter [4 mm, 0.2 μm polytetrafluoroethylene (PTFE), Thermo Fisher Scientific, Inc., Waltham, MA, U.S.A.], and pipetted into vials with glass inserts. The HT and BSA peak areas in the supernatant were compared to the peak areas of HTs and BSA in corresponding concentrations.

PP_50_ values were calculated by fitting dose–response curves (added HT concentration versus precipitated protein) in Origin (Origin 2015), using linear regression fitting and the calculated *x* from *y* function. For compounds **3** and **15**–**21**, the *x* axis was log_10_-transformed to obtain linear regression fitting. For compounds **1**, **4**, **5**, **8**, and **12**, only the linear part of the polynomial curves was used; this was possible because the PP_50_ concentrations were in the linear part of the dose–response curve. The fitted curves had *r*^2^ values of >0.95, except for compounds **1** and **14**, for which the *r*^2^ values were 0.89 and 0.90, respectively. The precipitated HT at PP_50_ was calculated by plotting the HT concentration versus precipitated HT, and data were fitted using the asymptotic regression model, except for compounds **2**, **4**, **5**, and **14**, in which the linear regression model was used. The calculated *x* from *y* function was used to determine the precipitated HT at the earlier determined PP_50_ value. The fitted curves had *r*^2^ values of >0.95, except for compounds **14**, **15** and **21**, for which the *r*^2^ values were 0.84, 0.91, and 0.93, respectively.

**Figure 3:**
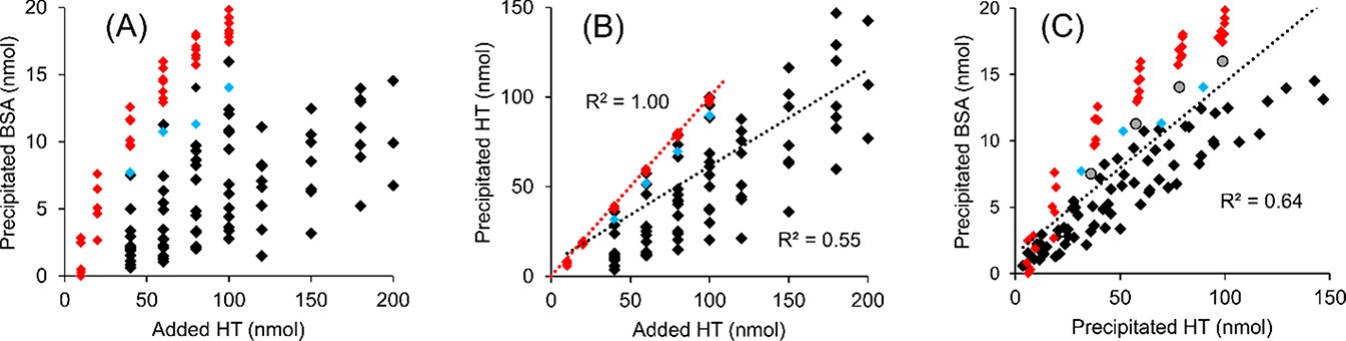
Correlation of (A) added HT versus precipitated BSA, (B) added HT versus precipitated HT, and (C) precipitated HT versus precipitated BSA when HT and BSA were incubated at pH 5 in various molar ratios. The black color indicates other monomeric structures than octagalloylglucose (compound **3** in Figure 1 and Table 1), which is indicated with gray circles in panel (C). The red color indicates other oligomeric structures than oenothein B (compound **14** in Figure 1 and Table 1), which is indicated with a light blue color. The amount of BSA in the reaction mixture was constant, 20 nmol, in all measurements.

The *r*^2^ values in the plots of Figures 3, 5, and 6 were generated in Excel (Microsoft Office 2016).

### UHPLC–DAD Analysis

Sample analysis was carried out with an Acquity UPLC system (Waters Corporation, Milford, MA, U.S.A.). The UPLC system consisted of a sample manager, a binary solvent manager, a column, and a diode array detector. The column used was a 100 × 2.1 mm inner diameter, 1.7 μm, Acquity UPLC BEH Phenyl column (Waters Corporation, Wexford, Ireland). The flow rate of the eluent was 0.5 mL min^–1^. The elution profile used two solvents, acetonitrile (A) and 0.1% aqueous formic acid (B): 0–0.5 min, 0.1% A in B; 0.5–5.0 min, 0.1–30% A in B (linear gradient); 5.0–5.1 min, 30–90% A in B (linear gradient); and 5.1–8.5 min, column wash and stabilization. Ultraviolet (UV) data (190–500 nm) were collected from 0 to 6 min.

## Results and Discussion

### General Trends in Precipitated Protein and Precipitated HT

In the present study, 21 HTs were selected on the basis of their PPC^24^ and studied in more detail to reveal how the HT structure affects the formation and characteristics of insoluble HT–protein complexes. BSA was used as model protein because it has often been used to measure the affinity of tannins toward proteins; this way, we could better correlate our results with previous studies. UHPLC–DAD was used to quantify both HT and BSA in the supernatant after HT–BSA precipitation, and from these data, the quantities of HT and BSA present in the precipitate were calculated. This facilitated the analysis of several hundred samples because no resolubilization of the precipitate was required after its separation from the supernatant. Also, the maximal absorbance values at 415 nm during the insoluble complex formation for each HT with BSA at the studied initial HT concentrations were measured with the turbidimetric method presented in the study by Engström et al.^24^ However, because the added HT versus absorbance were carefully studied the work by Engström et al.,^24^ this was less emphasized herein, and the focus was on added HT, precipitated protein, precipitated HT, their different correlations, and the complex compositions.

To find general, less HT-specific trends in the data, the dependency of precipitated BSA and precipitated HT on added HT was compared, first without compound categorization. The added HT versus precipitated BSA plot resulted in a weak correlation (Figure 3A), reflecting the expected variance in PPCs of the studied HTs. Comparison of monomers and oligomers further demonstrated the superior PPC of the oligomers in comparison to the monomers^14,21,22,24^ but also showed that the monomers had more variation in the added HT versus precipitated BSA than the oligomeric HTs studied.

The plot of added HT versus precipitated HT was best fitted by a linear regression model and resulted in a moderate positive relationship (Figure 3B). The amount of precipitated HT per added HT varied significantly, and in comparison of the activities of the monomers and oligomers, it was evident that, across the whole concentration range, the monomers had more compound-to-compound variation in their added HT versus precipitated HT plots than the oligomers. With regard to the oligomers, only oenothein B (**14**) deviated slightly from the other oligomers with lower precipitated HT values (Figure 3B), while for the other oligomers, the similarity of the added HT versus precipitated HT plots was so high that the *r*^2^ value for the linear regression was 1.00 (Figure 3B).

In the precipitated HT versus precipitated BSA plot, positive linear correlation was observed (Figure 3C). In general, the plot was quite similar to the added HT versus precipitated BSA plot (Figure 3A), with some variation in the plots of both the oligomeric and monomeric HTs. However, even the monomers expressed less variation in the precipitated HT versus precipitated BSA plot (Figure 3B) than in the added HT versus precipitated BSA plot (Figure 3A). In other words, the complex compositions of the insoluble HT–BSA complexes were less variable than the required added HT concentration to precipitate BSA. In addition, the results further confirmed that the oligomeric HTs, except oenothein B (**14**), were able to precipitate more BSA with the same amount of precipitated HT as the monomeric HTs, especially at higher precipitated HT values (Figure 3C). This emphasizes the importance of the multidentate nature of the oligomeric HTs when considering the ability to precipitate globular proteins, such as BSA.^20−22,24^ With regard to monomers, octagalloylglucose (**3**) precipitated more BSA with the same amount of HT present in the complexes (Figure 3C). This can be expected because octagalloylglucose has previously expressed high PPC in comparison to other monomeric HTs,^24^ and with the more flexible structure, it can cross-link BSA complexes more efficiently than the less flexible HHDP and HNTP esters or the smaller GGs with less flexible galloyls. This property becomes more prevalent especially at high tannin/protein ratios.

### Compound Categorization Based on Activity Trends

**Figure 4:**
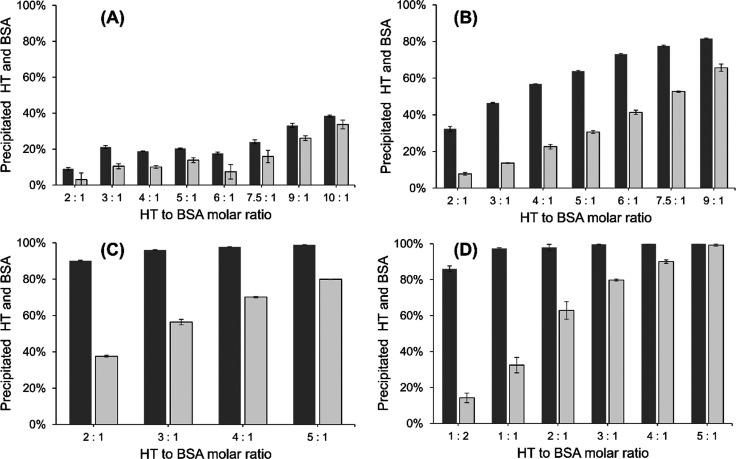
Examples of the four different profiles of precipitated HT (dark gray bars) and precipitated BSA (light gray bars) when incubated at various HT/BSA molar ratios: (A) 1,2,6-tri-*O*-galloyl-β-d-glucose (compound **1** in Figure 1 and Table 1), (B) geraniin (compound **7** in Figure 1 and Table 1, (C) octagalloylglucose (compound **3** in Figure 1 and Table 1), and (D) lambertianin C (compound **17** in Figure 2 and Table 1).

The individual plots of each 21 studied HTs showing the precipitated HT and precipitated BSA at each studied HT/BSA molar ratio (see Figures S1–S3 of the Supporting Information) could be divided into four different groups based on the overall trends. The first group contained trigalloylglucose (**1**), tellimagrandin I (**4**), vescalagin (**8**), and stachyurin (**12**), for which the amount of precipitated BSA as well as precipitated HT remained rather low (Figure 4A and Figures S1 and S2 of the Supporting Information), despite the increase of the HT/BSA molar ratio to 9 or 10. In the second group with 1,2-di-*O*-galloyl-4,6-HHDP-β-d-glucose (**5**), geraniin (**7**), castalagin (**9**), vescavaloninic acid (**10**), castavaloninic acid (**11**), and casuarinin (**13**), there was a clear and gradual increase of precipitated BSA and HT, although at low HT/BSA ratios, the precipitation was rather moderate (Figure 4B and Figures S1 and S2 of the Supporting Information). In the third group, including pentagalloylglucose (**2**), octagalloylglucose (**3**), punicalagin (**6**), and oenothein B (**14**), the precipitated HT increased rapidly and achieved a high rate (close to 90% HT precipitated at the highest HT/BSA molar ratio tested), but the amount of precipitated BSA remained below 80% (Figure 4C and Figures S1–S3 of the Supporting Information). The fourth group (Figure 4D and Figure S3 of the Supporting Information) contained all oligomeric ETs but not compound **14**, i.e., ETs **15**–**21**. The amount of precipitated HT achieved 100% rapidly, and the amount of precipitated BSA was above 80% at the highest HT/BSA ratios. However, none of the individual plots were identical, and to better compare the different HTs, parameters describing the efficacy to precipitate BSA were determined, including added HT, precipitated protein, precipitated HT, their different correlations, and the complex compositions.

### Effect of Added HT to Precipitated BSA and the Concentrations for Half-Maximal Protein Precipitation

**Figure 5:**
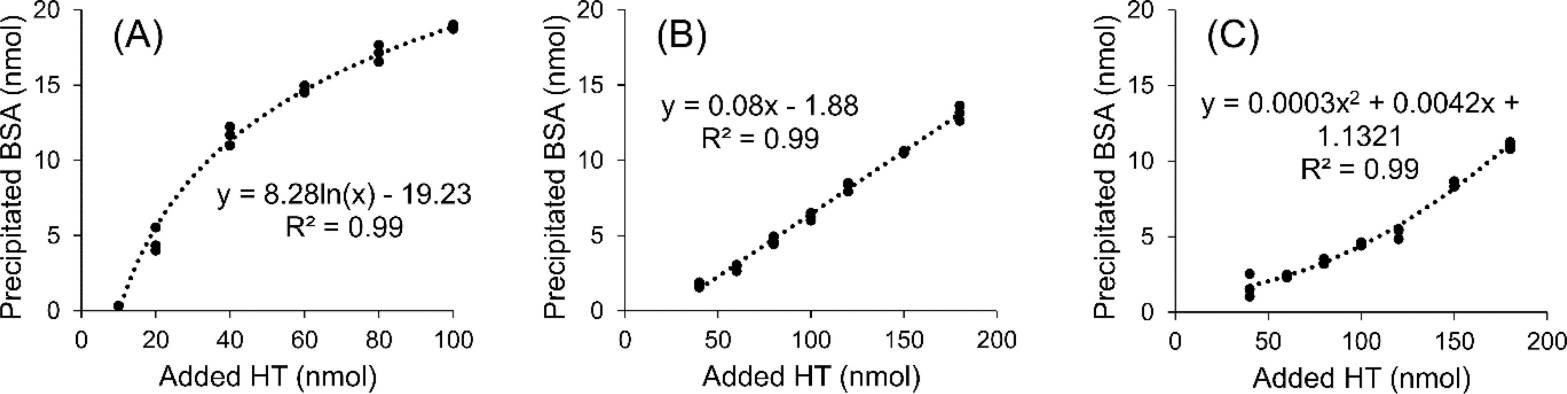
Examples of the three different profiles of added HT versus precipitated BSA plots: (A) agrimoniin (compound **19** in Figure 2 and Table 1), (B) geraniin (compound **7** in Figure 1 and Table 1), and (C) vescalagin (compound **8** in Figure 1 and Table 1).

When only the shapes of the added HT versus precipitated BSA plots were considered (Figure S4 of the Supporting Information), the studied HTs could be divided into three categories (Figure 5): for the oligomeric ETs, except oenothein B (**14**), and for octagalloylglucose (**3**), the plots were best fitted by logarithmic equations (Figure 5A and Figure S4 of the Supporting Information), indicating that, when increasing the HT/BSA molar ratios in the reaction mixture, the amount of precipitated BSA first quickly increases and then slowly levels off. For pentagalloylglucose (**2**), punicalagin (**6**), geraniin (**7**), castalagin (**9**), vescavaloninic acid (**10**), castavaloninic acid (**11**), casuarinin (**13**), and oenothein B (**14**), precipitated BSA increased linearly as the amount of added HT was increased (Figure 5B and Figure S4 of the Supporting Information). This was mostly due to the studied initial HT/BSA molar ratios, in which the maximum precipitation of BSA was not yet achieved for these HTs. The third group contained trigalloylglucose (**1**), tellimagrandin I (**4**), 1,2-di-*O*-galloyl-4,6-HHDP-β-d-glucose (**5**), vescalagin (**8**), and stachyurin (**12**), and for these, the curves were best fitted by second-order polynomial functions (Figure 5C and Figure S4 of the Supporting Information). At low initial HT/BSA ratios, little precipitation occurred, but after certain HT/BSA ratio, a linear increase was observed. The starting point of the linear increase was HT-specific. If considering a Hill slope often used to fit dose–response curves, the three groups above represented the different parts of a Hill slope. The steepness of the Hill slope is often used as a parameter describing activity, but as in our study, HT/BSA molar ratios in the initial reaction mixture resulting in the Hill slope could not be reached for all of the studied HTs. We next determined the concentrations for half-maximal protein precipitation (PP_50_), another commonly used parameter to describe PPC.^23,39^

**Figure 6:**
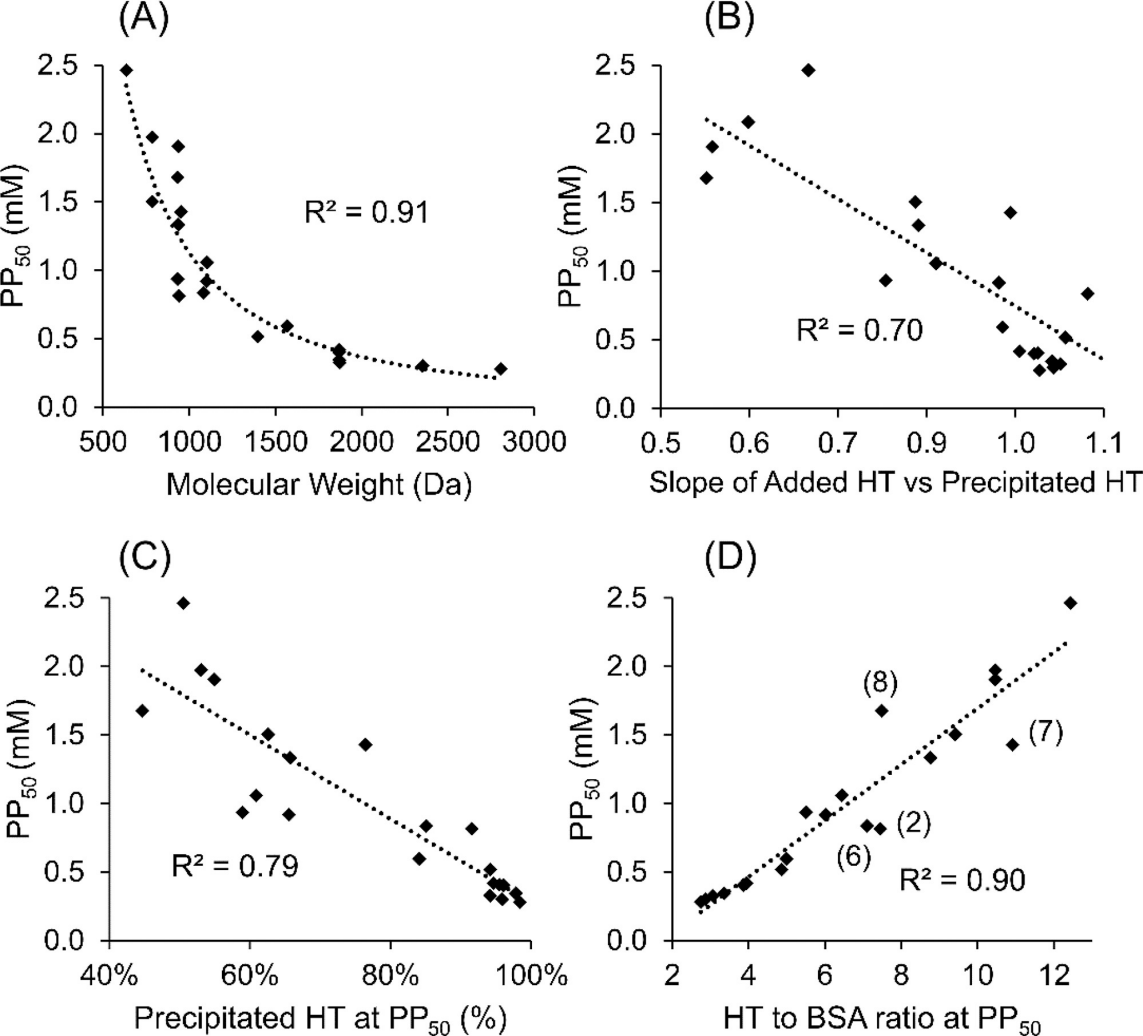
Correlation of (A) MW, (B) slope of added HT versus precipitated HT, (C) percentual precipitated HT at the added HT concentration at which 50% of added BSA precipitated (PP_50_), and (D) HT/BSA ratio at PP_50_ with the PP_50_ values. The four outliers, pentagalloylglucose (**2**), punicalagin (**6**), geranin (**7**), and vescalagin (**8**), are numbered.

To better compare the PPCs of the studied HTs, PP_50_ values indicating the required HT concentration to precipitate 50% of the BSA in the reaction solution were calculated for each HT (Table 1). As shown in numerous previous studies,^11,21,24^ the effect of the MW was significant (Figure 6A), and to some extent, higher MWs and, thus, the number of phenolic groups indicated lower PP_50_ values. However, as shown previously,^13,14,24^ especially at the MW area of 700–1200 Da, various PPCs were measured for HTs with MWs relatively close to each other. Thus, it is evident that, despite the significant effect of the HT MW on PPC, the other parameters, such as functional groups and overall flexibility of the structures, dominate over MW, especially when considering monomeric HTs. Therefore, a more detailed structure versus activity comparison was required to better explain the differences in the PPCs of the studied HTs.

**Table 1:** MWs, Required HT Concentrations for Half-Maximal Protein Precipitation (PP_50_), Precipitated HT (%) at PP_50_, HT/BSA Ratio in the Precipitate at PP_50_, and Slope (*k*) of Added HT versus Precipitated HT Plots

compound number	compound	MW (Da)	PP_50_ (mM)	precipitated HT (%) at PP_50_	HT/BSA ratio at PP_50_	*k*	95% confidence lower PP_50_	95% confidence upper PP_50_	95% confidence lower precipitated HT (%) at PP_50_	96% confidence upper precipitated HT (%) at PP_50_	95% confidence lower HT/BSA ratio at PP_50_	96% confidence upper HT/BSA ratio at PP_50_
**1**	1,2,6-tri-*O*-galloyl-β-d-glucose	636.47	2.46	51	12.4	0.70	2.23	2.70	48	53	11.9	13.0
**2**	1,2,3,4,6-penta-*O*-galloyl-β-d-glucose	940.67	0.81	92	7.5	1.11	0.71	0.92	91	92	7.4	7.5
**3**	octagalloylglucose	1396.99	0.52	94	4.9	1.05	0.49	0.54	94	95	4.8	4.9
**4**	tellimagrandin I	786.55	1.97	53	10.5	0.62	1.84	2.11	52	54	10.3	10.7
**5**	1,2-di-*O*-galloyl-4,6-HHDP-β-d-glucose	786.55	1.50	63	9.4	0.84	1.40	1.61	62	63	9.3	9.5
**6**	punicalagin	1084.71	0.84	85	7.1	1.08	0.78	0.89	84	86	7.0	7.2
**7**	geraniin	952.64	1.43	76	10.9	0.97	1.36	1.50	76	77	10.8	11.0
**8**	vescalagin	934.63	1.68	45	7.5	0.56	1.59	1.76	44	46	7.3	7.7
**9**	castalagin	934.63	0.93	59	5.5	0.80	0.88	0.99	58	60	5.4	5.6
**10**	vescavaloninic acid	1102.73	1.06	61	6.4	0.87	0.93	1.20	59	63	6.3	6.6
**11**	castavaloninic acid	1102.73	0.92	66	6.0	0.96	0.83	1.01	64	67	5.9	6.2
**12**	stachyurin	936.64	1.91	55	10.5	0.57	1.82	2.00	42	68	8.0	12.9
**13**	casuarinin	936.64	1.33	66	8.8	0.85	1.18	1.48	65	67	8.6	8.9
**14**	oenothein B	1569.08	0.59	84	5.0	0.96	0.40	0.79	83	85	4.9	5.1
**15**	oenothein A	2353.62	0.30	96	2.9	1.03	0.23	0.40	95	96	2.9	2.9
**16**	sanguiin H-6	1871.27	0.41	95	3.9	1.01	0.36	0.46	95	96	3.9	3.9
**17**	lambertianin C	2805.90	0.28	98	2.8	1.01	0.22	0.35	98	99	2.7	2.8
**18**	gemin A	1873.28	0.33	94	3.1	1.04	0.28	0.38	94	94	3.1	3.1
**19**	agrimoniin	1871.27	0.34	98	3.4	1.03	0.29	0.40	97	98	3.3	3.4
**20**	salicarinin A	1869.25	0.42	95	3.9	0.99	0.35	0.49	94	95	3.9	4.0
**21**	salicarinin B	1869.25	0.40	96	3.9	1.01	0.34	0.47	96	97	3.8	3.9

In general, the comparison of the PP_50_ values and structural characteristics of the studied HTs confirmed the conclusions made in the study by Engström et al.^24^ and various previous studies.^3,13,14^ In the present study, trigalloylglucose (**1**) with three galloyl groups was the smallest HT studied because it was the smallest galloyl glucose causing haze formation with BSA at the tested HT/BSA molar ratios. A comparison to the other galloylglucoses, penta- and octagalloylglucose (**2** and **3**, correspondingly), showed that the PPC increased, resulting in a decrease in PP_50_ values when the number of galloyl groups increased. In addition, the importance of the galloyl group at C1 was elucidated when comparing the PP_50_ values of tellimagrandin I (**4**) and 1,2-di-*O*-galloyl-4,6-HHDP-β-d-glucose (**5**).^24^ Within the oligomers, the selection of HTs for the present study allowed for the direct comparison of the effect of oxidative coupling only to oligomeric HTs gemin A (**18**) and agrimoniin (**19**). For these, no significant difference was observed in PP_50_ values, supporting the previous conclusions that the oligomerization partially diminishes the importance of different structural units that affect the PPCs of monomeric HTs with BSA.^22,24^

With regard to *C*-glycosidic ETs, the orientation of the hydroxyl group at the C1 position has been shown to affect both their physicochemical properties as well as bioactivities.^24,40−47^ Indeed, in the present study, significant differences were also observed in PP_50_ values in the *C*-glycosidic ETs **8**–**13**, **20**, and **21**. For the monomers, α orientation resulted in lower PP_50_ values and the effect was most significant between vescalagin (**8**) and castalagin (**9**, 0.75 units) than between stachyurin (**12**) and casuarinin (**13**, 0.58 units) and less significant between vescavaloninic acid (**10**) and castavaloninic acid (**11**, 0.15 units). For the dimers, salicarinin A (**20**) and salicarinin B (**21**), the difference was marginal but seemed to slightly favor βα orientation (**21**) over ββ orientation (**20**). As witnessed already in the study by Engström et al.,^24^ the orientation of the hydroxyl group at the C1 position of the *C*-glycosidic ETs also had an impact on the effect of other structural modifications. The addition of gallic acid to vescalagin (**8**) with β-hydroxyl at C1 to form vescavaloninic acid (**10**) decreased the PP_50_ value (0.62 units), while the addition of gallic acid to castalagin (**9**) to form castavaloninic acid (**11**) did not markedly affect the PP_50_ value (0.01 units decrease). The impact of the orientation of the hydroxyl group at the C1 position of the *C*-glycosidic ETs on the effect of oxidative coupling to PPC could be compared in the *C*-glycosidic HT pairs vescalagin (**8**) versus stachyurin (**12**) and castalagin (**9**) versus casuarinin (**13**), in which galloyl and HHDP groups of the former are oxidatively coupled to form the NHTP group of the latter. In both pairs, the formation of the NHTP group decreased the PP_50_. This is interesting because the opposite effect could be expected as a result of the rigidity brought to the structure when the NHTP group is formed. In our previous study,^24^ a similar effect was observed for castalagin versus casuarinin (**9** versus **13**); however, for vescalagin versus stachyurin (**8** versus **12**), the opposite effect was observed, and stachyurin had a slightly higher average PPC than vescalagin. A more detailed investigation of the results of the present study showed that this difference in the two studies was caused by the different activities of the two HTs at different parts of the added HT concentration range. While stachyurin (**12**) had higher PPC than vescalagin (**8**) at lower initial HT/BSA ratios, at higher HT/BSA ratios, vescalagin had higher PPC than stachyurin (Figure S2 of the Supporting Information). Thus, the structural rigidity brought to the HT structure by the NHTP group affects protein precipitation negatively at low HT/protein ratios, but when the concentration is increased, the effect is the opposite. This could be due to the more planar structure of NHTP containing vescalagin increasing the total surface area available for binding with BSA. However, with regard to castalagin (**9**) and casuarinin (**13**), castalagin was more efficient in precipitating BSA in the whole initial HT/BSA ratios studied. Altogether, these examples highlight that, in addition to the structural units present in a HT, also the intramolecular interactions resulting in possible differences in molecule shapes^32,48^ and the possible shielding of certain structural features are critical for the precipitation of BSA by HT. In addition, more specific interactions may occur between some of the studied HTs and BSA that cannot be interpreted by the more general structure–activity patterns. For example, certain structural change might allow or prevent the binding of a HT to a specific receptor site of the protein, which correspondingly might change the conformation of the protein, resulting in a different precipitation behavior.

The oligomers were superior to monomers in their capability to precipitate BSA, but the PP_50_ values of the trimers (**15** and **17**) did not decrease as much as expected by the corresponding decrease from monomers to dimers. This agrees with the previous studies indicating that the correlation between the tannin size and protein binding capacity may have an upper limit because the steric hindrance of large tannins may prevent access to binding sites.^15,24,49^ With regard to the dimers (excluding oenothein B, **14**), gemin A (**18**) and agrimoniin (**19**) with *m*-GOG linkage between the monomeric units had lower PPC values than sanguiin H-6 (**16**) with *m*-GOD and salicarinin A (**20**) and salicarinin B (**21**) with *m*-DOG linkage between the monomeric units. This indicates the beneficial effect of the more flexible *m*-GOG linkage, although the magnitude of the difference was subtle.

### Effect of Added HT to Its Precipitation

As seen from the individual plots (Figures S1–S3 of the Supporting Information), the trends of the added HT versus precipitated HTs were variable and could yield interesting additional information on the behavior of the HTs in the reaction mixture. Thus, we first plotted the added HT (nmol) against precipitated HT (nmol), and for all of the studied HTs, except trigalloylglucose (**1**), a strong positive linear correlation was obtained (Figure S5 of the Supporting Information), with *r*^2^ values of 0.97–1.0; for most of the HTs, the *r*^2^ values were 1.0, but for the weakest precipitators, *r*^2^ values were 0.97–0.98. This was caused by the lowest added HT concentration, where some bending was observed, suggesting that, if even lower added HT versus precipitated HTs were measured, the behavior of the plots would start resembling that of compound **1** in the lower added HT area. Thus, for compound **1**, the slope was calculated from the linear part at the higher added HT values.

When HT and protein are mixed in a reaction solution, soluble and/or insoluble complexes are formed.^4,50,51^ The relative proportion of the two depends upon both tannin and protein features as well as the reaction conditions; soluble complexes are favored when the protein concentration is in excess, whereas insoluble complexes are formed when tannins are present in excess.^4,13,51^ If the reaction conditions are kept otherwise identical and the only parameter changed is the added HT concentration, then there is a HT-dependent balance between the soluble and insoluble components. In a simplified scenario, if no cooperative binding would occur, the ratio of soluble and insoluble components would be constant, even if the added amount of HT would increase, as long as the amount of BSA in the reaction solution or number of binding sites in the BSA molecules would not start to limit this balance. However, the precipitation reaction is affected by cooperative binding, which includes (1) the HT–HT interactions when the HTs associate with the protein-bound HTs^15,52^ and (2) possible changes in the protein conformation during HT binding that enhance subsequent HT binding to other sites of the protein.^18,53^ Therefore, the added HT versus precipitated HT plot describes the tendency of the HT to move toward insoluble components, and the steepness of the slope describes how this changes when the amount of HT in the reaction mixture is increased and can be used to determine the significance of cooperative binding.

Plotting the slope values against PP_50_ values (Figure 6B) resulted in a linear negative correlation but not a perfect fit, indicating that, as can be expected, the two parameters are linked but also clear differences were witnessed. Interestingly, pentagalloylglucose (**2**) had the highest slope value of the studied HTs (Table 1), indicating a greater HT–HT interaction than even for the oligomeric ETs studied. Indeed, in previous studies, pentagalloylglucose has been shown to strongly self-associate,^54,55^ even forming a gel-like aqueous solution at room temperature at high concentrations.^55^ This indicates strong non-covalent cross-linking of the pentagalloylglucose molecules through hydrophobic interactions and hydrogen bonding.^56^ This can be linked to the strong hydrophobic character of pentagalloylglucose in comparison to the other studied HTs.^32,46,57^

In addition, octagalloylglucose (**3**) and punicalagin (**6**) had surprisingly high slope values in comparison to the other studied HTs (Table 1). This suggested a relatively high importance of the eight galloyl groups, the flexible structure of compound **3**, and the large-sized but non-flexible gallagyl group of compound **6** in cooperative binding. More surprisingly, for geraniin (**7**), with a DHHDP group in the structure, a much higher slope value was observed than expected on the basis of the PP_50_ value, indicating a strong tendency for the DHHDP group to self-associate or otherwise affect the cooperative binding. The central glucose of HTs is typically in ^4^C_1_ conformation, whereas in compound **7**, glucose is in an energetically less favorable ^1^C_4_ chair or intermediate skew-boat conformation,^58^ and thus, the galloyl group at O1 is exposed spatially in a different way, which could affect the cooperative binding positively. In agreement with the study by Engström et al.,^24^ the importance of the galloyl group at the O1 position was further indicated for the PP_50_ value but also for cooperative binding as the comparison of the structural isomers tellimagrandin I (**4**) and 1,2-di-*O*-galloyl-4,6-HHDP-β-d-glucose (**5**), the former with a higher PP_50_ value and less steep slope of the added HT versus precipitated HT slope.

With regard to *C*-glycosidic ETs **8**–**13**, the orientation of the hydroxyl group at the C1 position affected the slope values in a similar manner as was observed for the PP_50_ values; α orientation resulted in a steeper slope in all three pairs (Table 1; compound **8** versus compound **9**, compound **10** versus compound **11**, and compound **12** versus compound **13**). In the dimers salicarinin A (**20**) and salicarinin B (**21**), the difference was less pronounced but seemed to slightly favor βα orientation over ββ orientation similarly because it affected the PP_50_ value. Also, the addition of gallic acid to vescalagin (**8**) with β-hydroxyl and castalagin (**9**) with α-hydroxyl to form vescavaloninic acid (**10**) and castavaloninic acid (**11**), respectively, increased the slope value. For PP_50_ values, the positive effect was observed only for the former pair, indicating a different importance of the α-orientated hydroxyl group and additional gallic acid for the HT–BSA and HT–HT interactions. Interestingly, for vescalagin (**8**) versus stachyurin (**12**) and castalagin (**9**) versus casuarinin (**13**), no significant difference was observed in the slope values, although the formation of the NHTP group from the oxidative coupling of the galloyl and HHDP groups did result in decreased PPC (higher PP_50_ values). This indicates a greater importance of the galloyl + HHDP combination than the NHTP group in the protein precipitation reaction, but in cooperative binding, this does not have a big impact. With regard to the oligomeric ETs, oenothein B (**14**) had a lower slope steepness than the other oligomers, which also agreed with the difference in the PP_50_ values. For the other oligomers, the differences were subtle, as could be expected already on the basis of the similar PP_50_ values, indicating similar cooperative binding characteristics for all.

Although self-association of HTs is generally well-recognized,^15,52,59^ there are not many studies investigating the effect of the exact HT structure to self-association. However, a recent study by Virtanen et al.,^59^ in which HT–lipid interactions where studied by isothermal titration calorimetry, reported the heat profiles of different HTs when titrated into buffer solution without lipids. This progressively decreasing endothermic heat is attributed to the deaggregation process of the HTs and, thus, is linked to their degree of self-association.^15,52,59^ The profiles shown in the paper indicated strong self-association for oligomeric ETs, with lambertianin C having the highest observed heat rate in the buffer control measurement from the HTs present in our study. With regard to monomeric HTs, pentagalloylglucose and geraniin had high endothermic heat rates, while for vescalagin, trigalloylglucose, and tellimagrandin I, exothermic heat rates were observed. These results agreed with our findings regarding cooperative binding of these HTs. However, for punicalagin, Virtanen et al.^59^ showed a very low endothermic heat rate when titrated into the buffer solution, and this indicates that the cooperative-binging witnessed in our study stems at least partially from other interactions than self-association, for example, from the impact of punicalagin on the secondary structure of BSA.

Altogether, the data indicated significant differences in the importance of cooperative binding in the precipitation of BSA by different HTs, and thus, we calculated the percentage of precipitated HT at PP_50_ (Table 1) because these could yield insights on the operating efficiency of the HTs in precipitating BSA. As could be expected, the highest values were obtained for the best precipitators, the oligomeric ETs **15**–**21** and the monomeric HTs pentagalloylglucose (**2**) and octagalloylglucose (**3**). In addition, the low percentage of precipitated HT explained the high PP_50_ values of trigalloylglucose (**1**), tellimagrandin I (**4**), 1,2-di-*O*-galloyl-4,6-HHDP-β-d-glucose (**5**), vescalagin (**8**), and stachyurin (**12**); higher added HT was required to achieve PP_50_ simply because only 51–63% of the added HT was involved in the precipitation. Plotting the PP_50_ values against the percentage of HT precipitating at the PP_50_ concentration resulted in a negative linear correlation, with a *r*^2^ value of 0.79 (Figure 6C), again indicating that the percentage of HT precipitating explains PPC rather well but not fully.

Four of the studied HTs had lower PP_50_ values than expected on the basis of the precipitated HT (Figure 6C). Interestingly, these HTs were ETs **8**–**11** with an acyclic glucose core, all bearing a NHTP group in their structure. The other two *C*-glycosidic ETs with galloyl and HHDP groups instead of a NHTP group in the structure did not show the same divergency from the other HTs. Thus, this indicates that these four HTs differ from the other studied HTs regarding their mechanism of protein precipitation, for example, as a result of a more specific interaction with BSA.

### Complex Stoichiometries

The stoichiometries of HT–protein complexes have been studied in a few papers^16,30,32^ but not in a more comprehensive way to reveal possible differences between different subgroups of HT structures. To have a better understanding on how the complex stoichiometries in the precipitates were linked to the initial HT/BSA ratio and the HT structure, we calculated the HT/BSA ratios in the precipitates and plotted them against the HT/BSA ratio at the initial reaction mixture (Figure S6 of the Supporting Information). When the stoichiometries in the formed precipitates at the original HT/BSA ratios common to all tested HTs (2:1, 3:1, 4:1, and 5:1) are compared, the most obvious notion was again the similarity of the plots of the oligomeric HTs **15**–**21**, with a rather steady increase in HT/BSA ratio from 3:1 or 4:1 to 5:1. For oenothein B (**14**), the profile was otherwise rather similar as for the other oligomers, but at initial molar ratios of 4:1 and 5:1, the ratio in the precipitate was slightly higher than for the other oligomers, 6:1. With regard to monomeric HTs pentagalloylglucose (**2**), octagalloylglucose (**3**), vescalagin (**8**), and castalagin (**9**), the HT/BSA ratio in the precipitate increased rather steadily when the initial HT/BSA ratio was increased, however with varying ratios for each HT. This agreed with the previous studies, where the complex stoichiometry of pentagalloylglucose and BSA has been investigated,^16,30,32^ although in these studies, the range of the initial HT/BSA ratio was wider. For the other HTs, the HT/BSA ratio in the precipitate did not change much, even if the initial HT/BSA ratio was increased (**4** and **11**) or first increased and then leveled off or decreased (**1**, **5**–**7**, and **10**–**13**). When the additional initial HT/BSA ratios studied for certain HTs were included, these trends were further clarified. For example, for vescalagin (**8**), it was shown that the HT/BSA ratio in the precipitate started to decrease after a certain point, but the trend was not yet seen in the initial HT/BSA ratios common for all HTs. In addition, for geraniin (**7**), it was observed that the ratio in the precipitate did increase when the initial HT/BSA ratio was increased, although this was not seen from the initial HT/BSA ratios common for all HTs. For some of the oligomeric HTs, additional lower HT/BSA ratios (1:2 and 1:1) were studied (**15**, **17**–**19**, and **21**). These additional determinations demonstrated that, for the dimers gemin A (**18**), agrimoniin (**19**), and salicarinin B (**21**), very high HT/BSA ratios in the precipitates were obtained when the initial HT/BSA ratio was 1:2. For the trimers (**15** and **17**), this phenomenon was not seen as a result of the high PPC already at the lowest initial HT/BSA ratios studied (>10% BSA precipitated already at the lowest studied initial HT/BSA ratio studied). This demonstrates a high tendency of the dimers to form insoluble complexes with BSA, although very low amounts of BSA precipitated at this initial HT/BSA ratio, indicating significant self-association and consequently cooperative binding of the oligomers. When the HT/BSA ratio was 1:2, the dimers did not induce significant precipitation, partially as a result of the lack of dimers effectively cross-linking BSA molecules, in which the larger trimers were already able to achieve at these low molar ratios.

The data demonstrated well that, even if the overall trends in the HT/BSA ratios in the precipitates followed certain logic reflecting the PPC of the HTs, an explanation of the results was not straightforward and similar HT/BSA ratios could be observed with HTs expressing very different PPCs. For example, vescalagin (**8**) with a low PPC had quite a similar profile at initial HT/BSA ratios of 2:1, 3:1, 4:1, and 5:1 as the more effective protein precipitators (**2**, **3**, and **14**–**21**). Thus, the two parameters should be evaluated side by side to fully interpret them. However, if the amount of precipitated protein would be the same for all HTs as it is at PP_50_, then the HT/BSA ratio in the precipitate is more explicit, demonstrating the required HT/BSA ratio in the precipitate. When the stoichiometries of the precipitates at PP_50_ were calculated for the 21 HTs (Table 1), a wide range of complex compositions at PP_50_ were obtained. In general, the better protein precipitator the HT, the lower the HT/BSA ratio at PP_50_. For the best precipitators, the oligomeric HTs (excluding oenothein B, **14**), the ratio was 2.8–3.9. Thereafter, the HT/BSA ratio in the complexes increased rather linearly with the increase of PPC, and for the HTs with the lowest PPC from the studied HTs, the HT/BSA ratios in the precipitates at PP_50_ were 10.5–12.3. It is difficult to compare these results to previous studies as a result of the very different experimental approaches, but in the study by Kawamoto et al.,^16,30^ the HT/BSA ratio at PP_50_ was on the same level as in the present study for pentagalloylglucose, while for trigalloylglucose, the HT/BSA ratio at PP_50_ was higher than observed in the present study (>20). With regard to the trigalloylglucoses, in the study by Kawamoto et al.,^16,30^ synthesized galloylglucoses had galloyl groups at C2, C3, and C6 or C2, C3, and C4, while in present study, the distribution of the galloyl groups in naturally occurring trigalloylglucose was C1, C2, and C6. In addition, Kawamoto et al.^16,30^ used C1-methylated galloylglucoses, which makes the comparison even more difficult.

When the HT/BSA ratios at the PP_50_ were plotted against the PP_50_ values, a strong positive correlation was obtained (Figure 6D), which further confirmed that the complex compositions of the precipitates are linked to the PPC of the studied HT. However, there were four HTs that were considered as outliers in the plot: three outliers with a higher HT/BSA ratio and one HT with a lower HT/BSA ratio in the precipitate than expected on the basis of the PP_50_ values. The former mentioned were pentagalloylglucose (**2**), punicalagin (**6**), and geranin (**7**). In addition, these HTs had surprisingly high added HT versus precipitated HT slope values, with pentagalloylglucose with the highest slope in comparison to other monomers and oligomers. This further emphasizes the importance of cooperative binding of these three HTs; they have a high tendency to precipitate with BSA but are not yet precipitating BSA as efficiently as, for example, the oligomeric HTs studied. This is likely due to the smaller size and structural rigidity in comparison to the oligomers and inability to efficiently cross-link between BSA molecules to enhance the precipitation reaction. The one HT with higher PP_50_ values than the other studied HTs based on the HT/BSA ratio at PP_50_ was vescalagin (**8**). It also had the lowest precipitated HT (%) at PP_50_ and the lowest slope of all added HT versus precipitated HT curves. As discussed earlier in the text, vescalagin had a lower PP_50_ value than expected on the basis of the precipitated HT. This deviation from the other HTs studied was also reflected in the HT/BSA ratio at PP_50_ versus PP_50_ plot. Altogether, the results showed that, with regard to the monomeric HTs, the complex compositions are unique and HT-specific and affected by the same features that affect the PPC of the HTs. With regard to oligomeric HTs, the compositions of the precipitates were more uniform, similarly as observed for both the PPC and slopes of added versus precipitated HT.

To conclude, the results obtained in the present study indicated a clear correlation between structural features of HTs and the characteristics of the formed insoluble HT–BSA complexes. On the basis of the results, it seems that the greater tendency of certain HTs to form insoluble complexes when mixed with protein is partially linked to the higher self-association and consequently stronger cooperative binding of these HTs with BSA. However, on the other hand, our data demonstrated that, for certain HTs, at least at the studied initial HT/BSA ratios, 100% binding of the HT to BSA is never achieved. This again points toward a higher tendency to the soluble phase and can at least partially be explained by weaker affinity toward BSA but also the lower tendency to self-associate. Altogether, our results clearly established that the interactions between HTs and proteins, at least BSA, are highly HT-specific regarding the protein precipitation as well as the stoichiometry of the precipitation reaction. To continue to unravel the HT–protein complexation, the next obvious step would be to investigate the stabilities of the HTs and the formed insoluble HT–protein complexes in different experimental conditions. This would further increase the understanding of the mechanisms behind the various bioactivities that HTs express and facilitate their potential use in different tannin-based applications.

## Supporting Information Available

Studied 21 HTs with information on the original plant material, purity by UPLC at 280 nm, and ESI–MS identification (S1 Appendix), individual plots of each studied HT showing the precipitated HT and BSA at each studied initial HT/BSA molar ratio (Figures S1–S3), individual plots of each studied HT showing the added HT versus precipitated BSA (Figure S4), individual plots of each studied HT showing the added HT versus precipitated HT (Figure S5), and individual plots of each studied HT showing the initial HT/BSA ratio versus the HT/BSA ratio in the precipitate (Figure S6) (PDF)

## Abbreviations Used

BSA: bovine serum albumin
DHHDP: dehydrohexahydroxydiphenoyl
*m*-DOG: valoneoyl group
2× *m*-DOG: macrocyclic structure
ESI: electrospray ionization
ET: ellagitannin
G: galloyl group
*m*-GOD: sanguisorboyl group
*m*-GOG: dehydrodigalloyl group
HHDP: hexahydroxydiphenoyl
HT: hydrolyzable tannin
MS: mass spectrometry
MW: molecular weight
NHTP: nonahydroxytriphenoyl
PP_50_: concentration for half-maximal protein precipitation
PPC: protein precipitation capacity
TQ: triple quadrupole
UPLC: ultra-high-performance chromatography
