# Supplementary material for: Influence of the Hydrolyzable Tannin Structure on the Characteristics of Insoluble Hydrolyzable Tannin–Protein Complexes
Source: J Agric Food Chem. 2022 Jun 16;70(41):13036–48. doi: 10.1021/acs.jafc.2c01765 (PMC9585579; doi:10.1021/acs.jafc.2c01765)
Supplement: Supplementary file 1 — jf2c01765_si_001.pdf [file jf2c01765_si_001.pdf]

Supporting Information for “Influence of the Hydrolyzable Tannin Structure on the Characteristics of Insoluble Hydrolyzable Tannin–Protein Complexes”

Marica T. Engström, Valtteri Virtanen and Juha-Pekka Salminen

Natural Chemistry Research Group, Department of Chemistry, University of Turku, FI-20014, Turku, Finland

*Correspondence: mtengs@utu.fi, Phone: +358 29 450 3168

S1 Appendix. The studied 21 hydrolyzable tannins with information on the original plant material, the purity by UHPLC-DAD at 280 nm and the ESI-MS identification

Trigalloylglucose (1) was isolated from *Betula pubescens* leaves as described in Salminen et al. (2001); UHPLC-DAD purity at 280 nm: 99.6%; ESI-MS identification: *m/z* at 635 $[M–H]^-$; identified earlier by Salminen et al. (2001).

Pentagalloylglucose (**2**) was purified from tannic acid purchased from J.T. Baker (Denventer, Holland) as described in Salminen et al. (2001); UHPLC-DAD purity at 280 nm: 99.8%; ESI-MS identification: *m/z* at 939 $[M–H]^-$; identified earlier by Salminen et al. (1999, 2001), Salminen and Karonen (2011).

Octagalloylglucose (**3**) was isolated from tannic acid purchased from J.T. Baker (Denventer, Holland); UHPLC-DAD purity at 280 nm: 94.3%; ESI-MS identification: *m/z* at 1395 $[M–H]^-$; identified earlier by Salminen et al. (2001), Salminen and Karonen (2011).

Tellimagrandin I, monomeric ET (**4**), was isolated from *Filipendula ulmaria* inflorescence; UHPLC-DAD purity at 280 nm: 97.5%; ESI-MS identification: *m/z* at 785 $[M–H]^-$; identified earlier by Salminen et al. (2001), Moilanen et al. (2013).

1,2-digalloyl-4,6-HHDP-glucose (**5**) was isolated from *Geranium sylvaticum* leaves; UHPLC-DAD purity at 280 nm: 97.8%; ESI-MS –identification: *m/z* at 785 $[M–H]^-$; identified earlier by Tuominen et al. 2013.

Punicalagin (6) was isolated from *Terminalia chebula* fruits; UHPLC-DAD purity at 280 nm: 97.6%; ESI-MS –identification: *m/z* at 1083 $[M–H]^-$, identified earlier from *Terminalia* by Pfundstein et al. (2010). The structure was confirmed by NMR.

Geraniin (**7**) was isolated from *Geranium sylvaticum* leaves; UHPLC-DAD purity at 280 nm: 98.3%; ESI-MS –identification: *m/z* at 951 $[M–H]^–$, *m/z* at 933 $[M–H_2O–H]^–$, *m/z* at 301 $[\text{ellagic acid}–H]^–$; identified earlier by Tuominen et al. (2013).

Vescalagin (**8**) was isolated from *Lythrum salicaria* flowers and leaves; UHPLC-DAD purity at 280 nm: 94.1%; ESI-MS –identification: *m/z* at 933 $[M–H]^–$, *m/z* at 915 $[M–H_2O–H]^–$, *m/z* at 466 $[M–2H]^{2–}$, *m/z* at 457 $[M–H_2O –2H]^{2–}$, *m/z* at 301 $[\text{ellagic acid}–H]^–$; identified earlier by Rauha et al. (2001), Salminen et al. (2004), Moilanen and Salminen (2008), Moilanen et al. (2013).

Castalagin (**9**) was isolated from *Lythrum salicaria* flowers and leaves; UHPLC-DAD purity at 280 nm: 96.8%; ESI-MS –identification: *m/z* at 933 $[M–H]^–$, *m/z* at 466 $[M–2H]^{2–}$, *m/z* at 301 $[\text{ellagic acid}–H]^–$; identified earlier by Rauha et al. (2001), Salminen et al. (2004), Moilanen and Salminen (2008), Moilanen et al. (2013).

Vescavaloninic acid (**10**) was isolated from *Quercus robur* acorns; UHPLC-DAD purity at 280 nm: 95.6%; ESI-MS –identification: *m/z* at 1101 $[M–H]^–$, *m/z* at 1083 $[M–H_2O–H]^–$, *m/z* at 528 $[M–COOH–H]^{2–}$, *m/z* at 519 [$[M–H_2O–COOH–H]^{2–}$, *m/z* at 301 $[\text{ellagic acid}–H]^–$; identified earlier by Yarnes et al. (2006, 2008), Moilanen and Salminen (2008).

Castavaloninic acid (**11**) was isolated from *Quercus robur* acorns; UHPLC-DAD purity at 280 nm: 94.1%; ESI-MS –identification: *m/z* at 1101 $[M–H]^–$, *m/z* at 528 $[M–COOH–H]^{2–}$; identified earlier by Yarnes et al. (2006, 2008), Moilanen and Salminen (2008).

Stachyurin (**12**) was isolated from *Hippophaë rhamnoides* leaves; UHPLC-DAD purity at 280 nm: 94.4%; ESI-MS –identification: *m/z* at 935 $[M–H]^–$; identified earlier by Moilanen and Salminen (2008), Moilanen et al. 2013.

Casuarinin (**13**) was isolated from *Hippophaë rhamnoides* leaves; UHPLC-DAD purity at 280 nm: 88.9%; ESI-MS –identification: *m/z* at 935 $[M–H]^{–}$; identified earlier by Moilanen and Salminen (2008), Moilanen et al. 2013.

Oenothein B, dimeric ET (**14**), was isolated from *Epilobium angustifolium* inflorescence; UHPLC-DAD purity at 280 nm: 98.2%; ESI-MS –identification: *m/z* at 783 $[M–2H]^{2–}$, identified earlier by Moilanen and Salminen (2008), Moilanen et al. (2013), Baert et al. (2015).

Oenothein A, trimeric ET (**15**), was isolated from *Epilobium angustifolium* inflorescence; UHPLC-DAD purity at 280 nm: 95.7%; ESI-MS –identification: *m/z* at 1175 $[M–2H]^{2–}$, identified earlier by Karonen et al. (2010) and Baert et al. (2015).

Sanguiin H-6 (**16**) was isolated from *Rubus idaeus* leaves; UHPLC-DAD purity at 280 nm: 92.6%; ESI-MS –identification: *m/z* at 934 $[M–2H]^{2–}$, *m/z* at 301 [ellagic acid–H]$^{–}$; identified earlier by Moilanen and Salminen (2008).

Lambertianin C (**17**) was isolated from *Rubus idaeus* leaves; UHPLC-DAD purity at 280 nm: 96.9%; ESI-MS –identification: *m/z* at 934 $[M–3H]^{3–}$, *m/z* at 301 [ellagic acid–H]$^{–}$; identified earlier by Moilanen and Salminen (2008).

Gemin A (**18**) was isolated from *Geum urbanum* leaves; UHPLC-DAD purity at 280 nm: 98.2%; ESI-MS –identification: *m/z* at 935 $[M–2H]^{2–}$, *m/z* at 301 [ellagic acid–H]$^{–}$; identified earlier by Moilanen and Salminen (2008).

Agrimoniin (**19**) was isolated from *Potentilla anserina* leaves; UHPLC-DAD purity at 280 nm: 99.0%; ESI-MS –identification: *m/z* at 934 $[M–2H]^{2–}$, *m/z* at 301 [ellagic acid–H]$^{–}$; identified by Moilanen and Salminen (2008), Moilanen et al. (2013).

Salicarinin A (**20**) was isolated from *Lythrum salicaria* flowers and leaves; UHPLC-DAD purity at 280 nm: 96.8%; ESI-MS –identification: *m/z* at 933 $[M–2H]^{2–}$, 924 $[M–H_2O –2H]^{2–}$, 915 $[M–2H_2O –2H]^{2–}$,

m/z at 1867 $[M–H]^{–}$, identified earlier by Piwowarski and Kiss (2013). The structure was confirmed by NMR spectroscopy.

Salicarinin B (21) was isolated from *Lythrum salicaria* flowers and leaves; UHPLC-DAD purity at 280 nm: 95.8%; ESI-MS –identification: *m/z* at 933 $[M–2H]^{2–}$, 924 $[M–H_2O –2H]^{2–}$, *m/z* at 1867 $[M–H]^{–}$, identified earlier by Piwowarski and Kiss (2013). The structure was confirmed by NMR spectroscopy.

References:

Baert N, Karonen M, Salminen J-P. Isolation, characterisation and quantification of the main oligomeric macrocyclic ellagitannins in Epilobium angustifolium by ultra-high performance chromatography with diode array detection and electrospray tandem mass spectrometry. J Chromatogr A. 2015;1419: 26–36.

Karonen M, Parker J., Agrawal A., Salminen JP. First evidence of hexameric and heptameric ellagitannins in plants detected by liquid chromatography/electrospray ionisation mass spectrometry. Rapid Commun. Mass Spectrom. 2010;24: 3151–3156.

Moilanen J, Salminen J-P. Ecologically neglected tannins and their biologically relevant activity: chemical structures of plant ellagitannins reveal their in vitro oxidative activity at high pH. Chemoecology. 2008;18: 73–83.

Moilanen J, Sinkkonen J, Salminen J-P. Characterization of bioactive plant ellagitannins by chromatographic, spectroscopic and mass spectrometric methods. Chemoecology. 2013;23: 165–179.

Moilanen J, Koskinen P, Salminen J-P. Distribution and content of ellagitannins in Finnish plant species. Phytochemistry. Elsevier Ltd; 2015;116: 188–197.

Piwowarski JP, Kiss AK. C-glucosidic ellagitannins from Lythri herba (European Pharmacopoeia): Chromatographic profile and structure determination. Phytochem. Anal. 2013;24: 336–348.

Rauha J-P, Wolfender J-L, Salminen J-P, Pihlaja K, Hostettmann K, Vuorela H. Characterization of the Polyphenolic Composition of Purple Loosestrife (Lythrum salicaria). Zeitschrift für Naturforsch C. 2001;56: 13–20.

Tuominen A, Toivonen E, Mutikainen P, Salminen J-P. Defensive strategies in Geranium sylvaticum. Part 1: Organ-specific distribution of water-soluble tannins, flavonoids and phenolic acids. Phytochemistry. 2013;95: 394–407.

Salminen JP, Ossipov V, Loponen J, Haukioja E, Pihlaja K. Characterization of hydrolysable tannins from leaves of Betula pubescens by high-performance liquid chromatography – mass spectrometry. J. Chrom. A 1999;864: 283–291.

Salminen JP., Ossipov V, Haukioja E, Pihlaja K. Seasonal variation in the content of hydrolysable tannins in leaves of Betula pubescens. Phytochemistry 2001;57: 15–22.

Salminen JP, Karonen M. Chemical ecology of tannins and other phenolics: we need a change in approach. Funct. Ecol. 2011;25: 325–338.

Yarnes CT, Boecklen WJ, Tuominen K, Salminen JP. Defining phytochemical phenotypes: size and shape analysis of phenolic compounds in oaks (Fagaceae, Quercus) of the Chihuahuan Desert. Can. J. Bot. 2006;84: 1233–1248.

Yarnes CT, Boecklen WJ, Salminen JP. No simple sum: seasonal variation in tannin phenotypes and leaf-miners in hybrid oaks. Chemoecology 2008;18: 39–51.

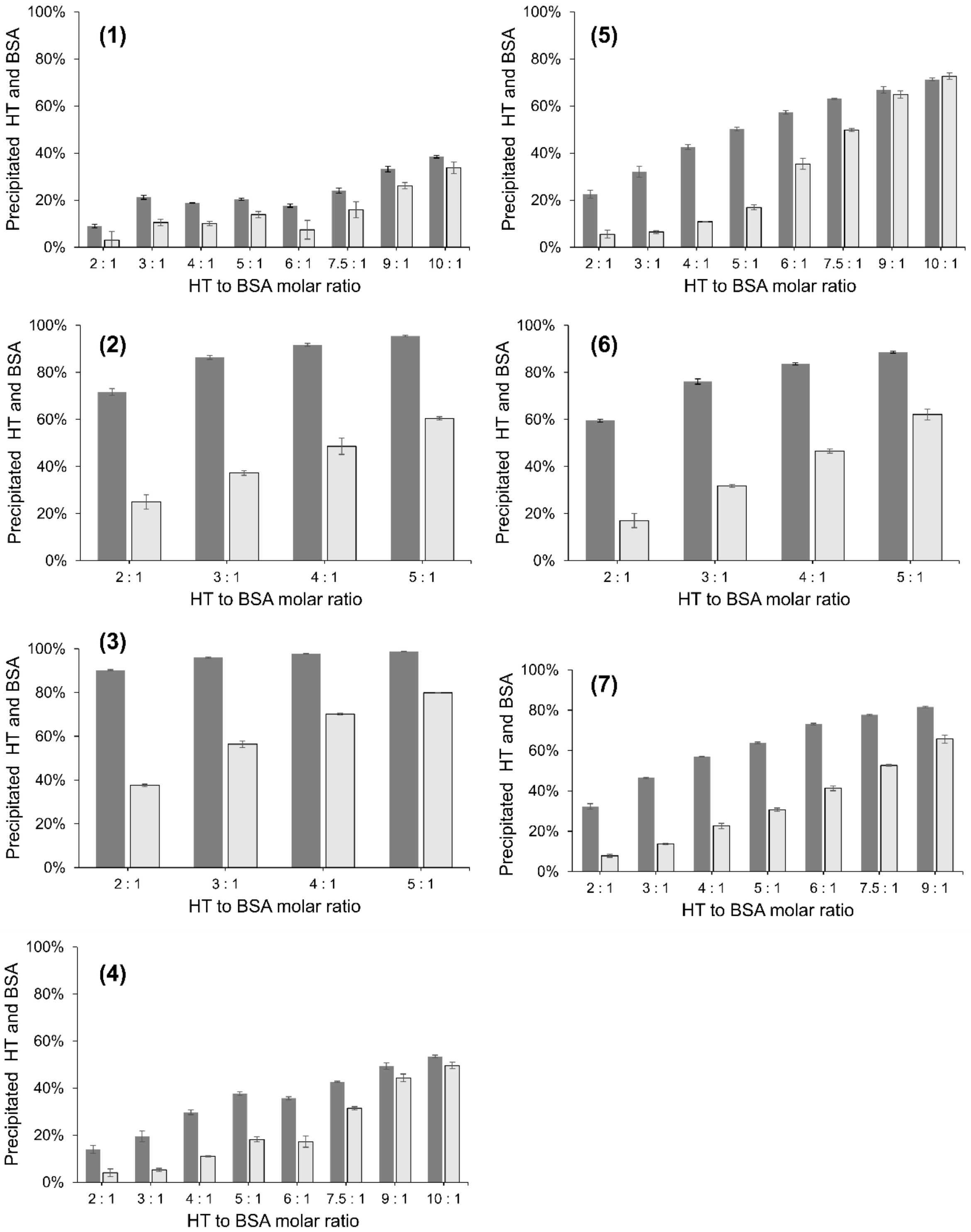

Figure S1. The individual plots of each studied hydrolyzable tannin (HT) showing the precipitated HT (dark grey bars) and bovine serum albumin (BSA) (light grey bars) at each studied initial HT to BSA molar ratio. (**1**) 1,2,6-tri-*O*-galloyl-β-D-glucose, (**2**) 1,2,3,4,6-penta-*O*-galloyl-β-D-glucose, (**3**) octagalloylglucose, (**4**) tellimagrandin I, (**5**) 1,2-di-*O*-galloyl-4,6-HHDP-β-D-glucose, (**6**) punicalagin and (**7**) geraniin.

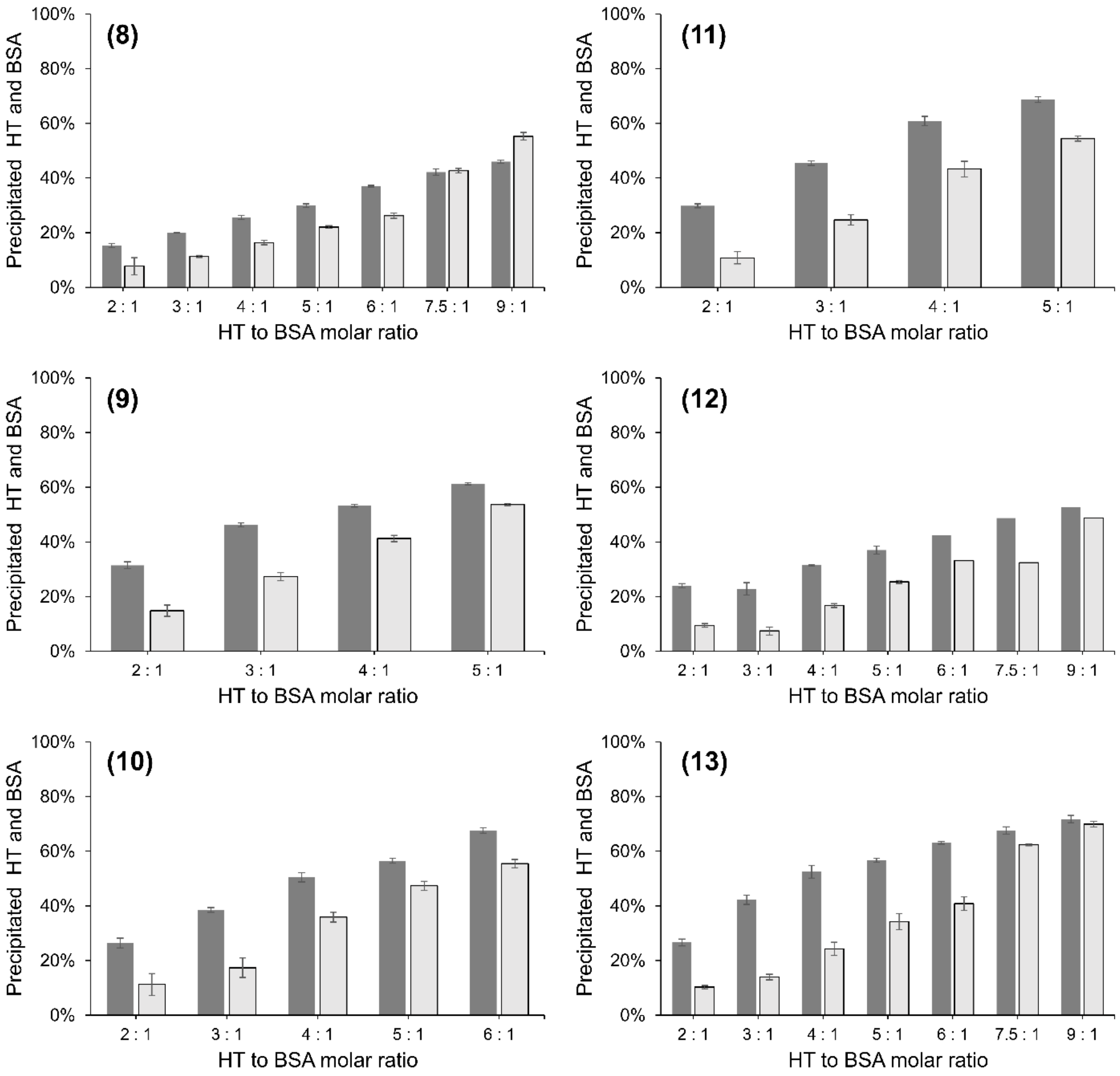

Figure S2. The individual plots of each studied *C*-glycosidic monomeric hydrolyzable tannin (HT) showing the precipitated HT (dark grey bars) and bovine serum albumin (BSA) (light grey bars) at each studied initial HT to BSA molar ratio. (**8**) vescalagin, (**9**) castalagin, (**10**) vescavaloninic acid, (**11**) castavaloninic acid, (**12**) stachyurin and (**13**) casuarinin.

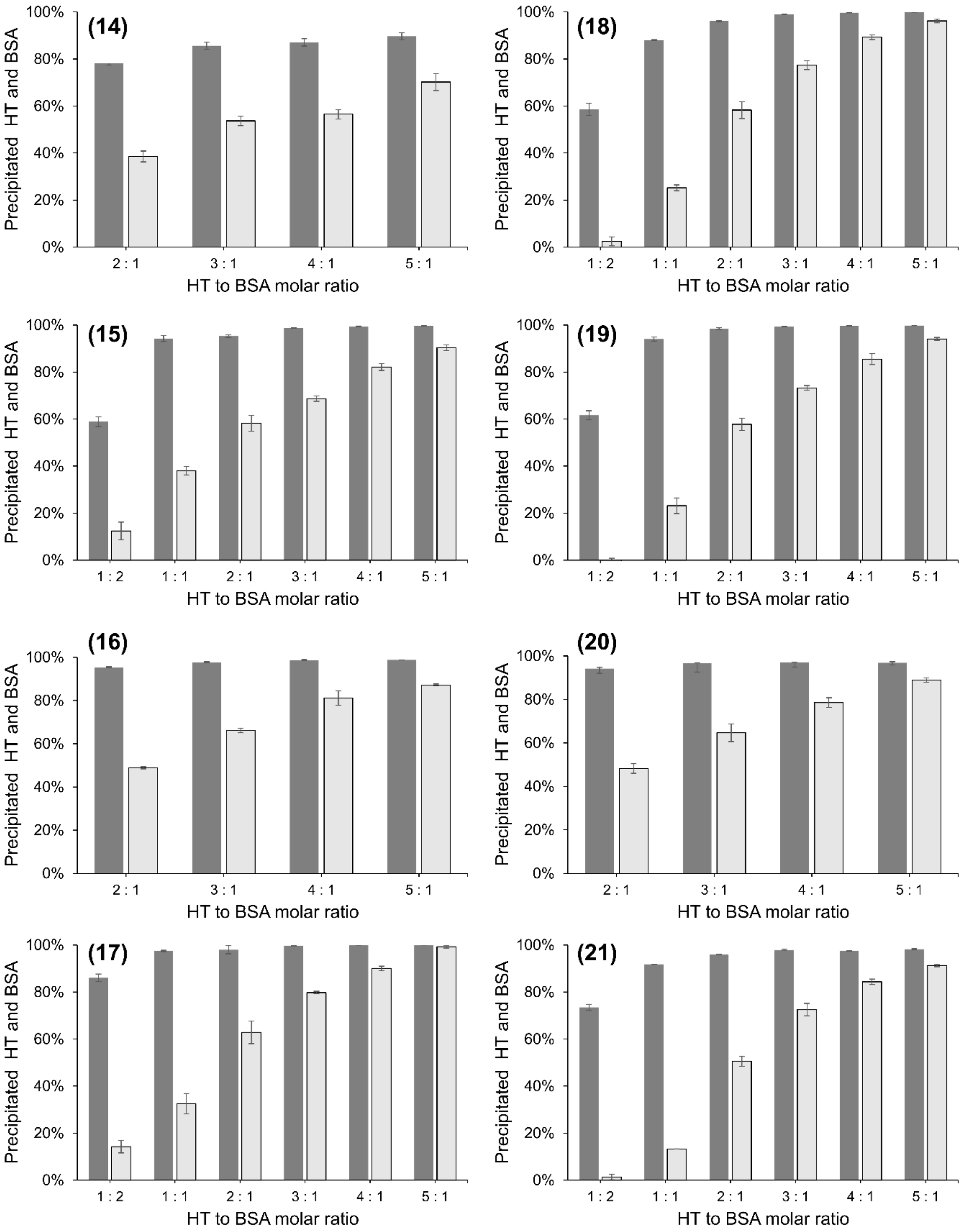

Figure S3. The individual plots of each studied oligomeric hydrolyzable tannin (HT) showing the precipitated HT (dark grey bars) and bovine serum albumin (BSA) (light grey bars) at each studied initial HT to BSA molar ratio. (**14**) oenothein B, (**15**) oenothein A, (**16**) sanguiin H-6, (**17**) lambertianin C, (**18**) gemin A, (**19**) agrimoniin, (**20**) salicarinin A and (**21**) salicarinin B.

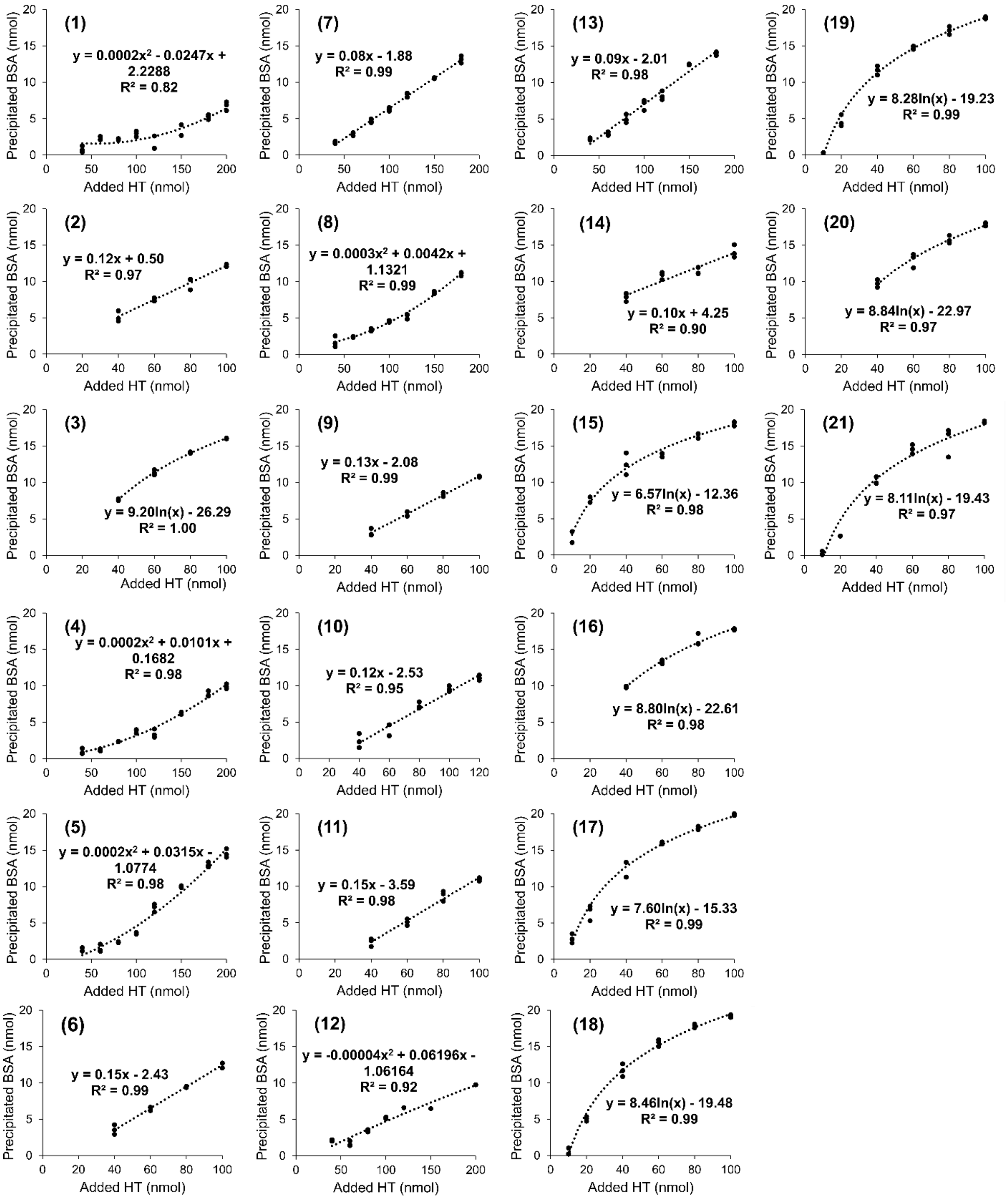

Figure S4. The individual plots of each studied hydrolyzable tannin (HT) showing the added HT versus precipitated bovine serum albumin (BSA). (1) 1,2,6-tri-O-galloyl-β-D-glucose, (2) 1,2,3,4,6-penta-O-galloyl-β-D-glucose, (3) octagalloylglucose, (4) tellimagrandin I, (5) 1,2-di-O-galloyl-4,6-HHDP-β-D-glucose, (6) punicalagin, (7) geraniin, (8) vescalagin, (9) castalagin, (10) vescavaloninic acid, (11) castavaloninic acid, (12) stachyurin, (13) casuarinin, (14) oenothein B, (15) oenothein A, (16) sanguiin H-6, (17) lambertianin C, (18) gemin A, (19) agrimoniin, (20) salicarinin A and (21) salicarinin B.

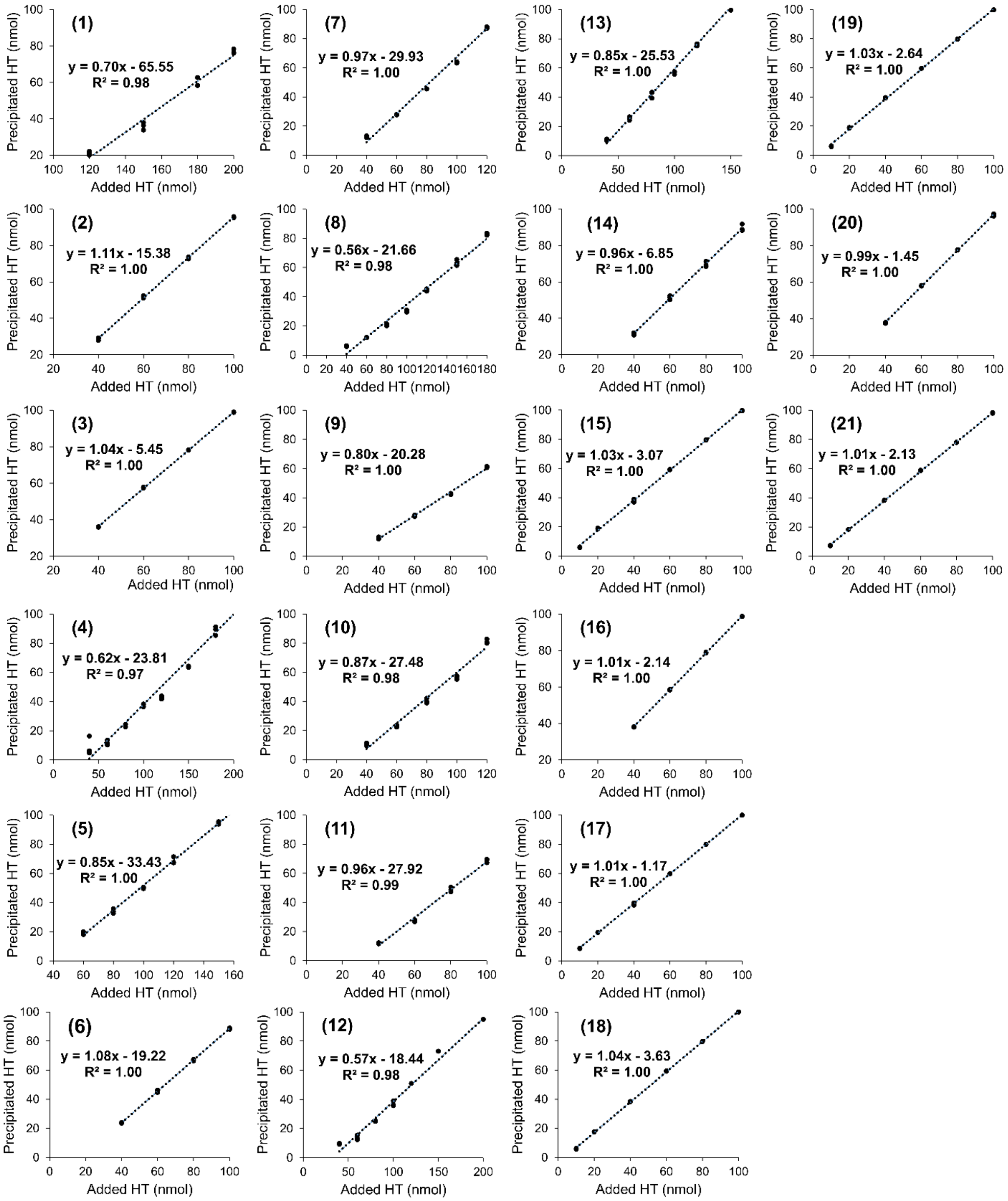

Figure S5. The individual plots of each studied hydrolyzable tannin (HT) showing the added HT versus precipitated HT. (**1**) 1,2,6-tri-*O*-galloyl-β-D-glucose, (**2**) 1,2,3,4,6-penta-*O*-galloyl-β-D-glucose, (**3**) octagalloylglucose, (**4**) tellimagrandin I, (**5**) 1,2-di-*O*-galloyl-4,6-HHDP-β-D-glucose, (**6**) punicalagin, (**7**) geraniin, (**8**) vescalagin, (**9**) castalagin, (**10**) vescavaloninic acid, (**11**) castavaloninic acid, (**12**) stachyurin, (**13**) casuarinin, (**14**) oenothein B, (**15**) oenothein A, (**16**) sanguiin H-6, (**17**) lambertianin C, (**18**) gemin A, (**19**) agrimoniin, (**20**) salicarinin A and (**21**) salicarinin B.

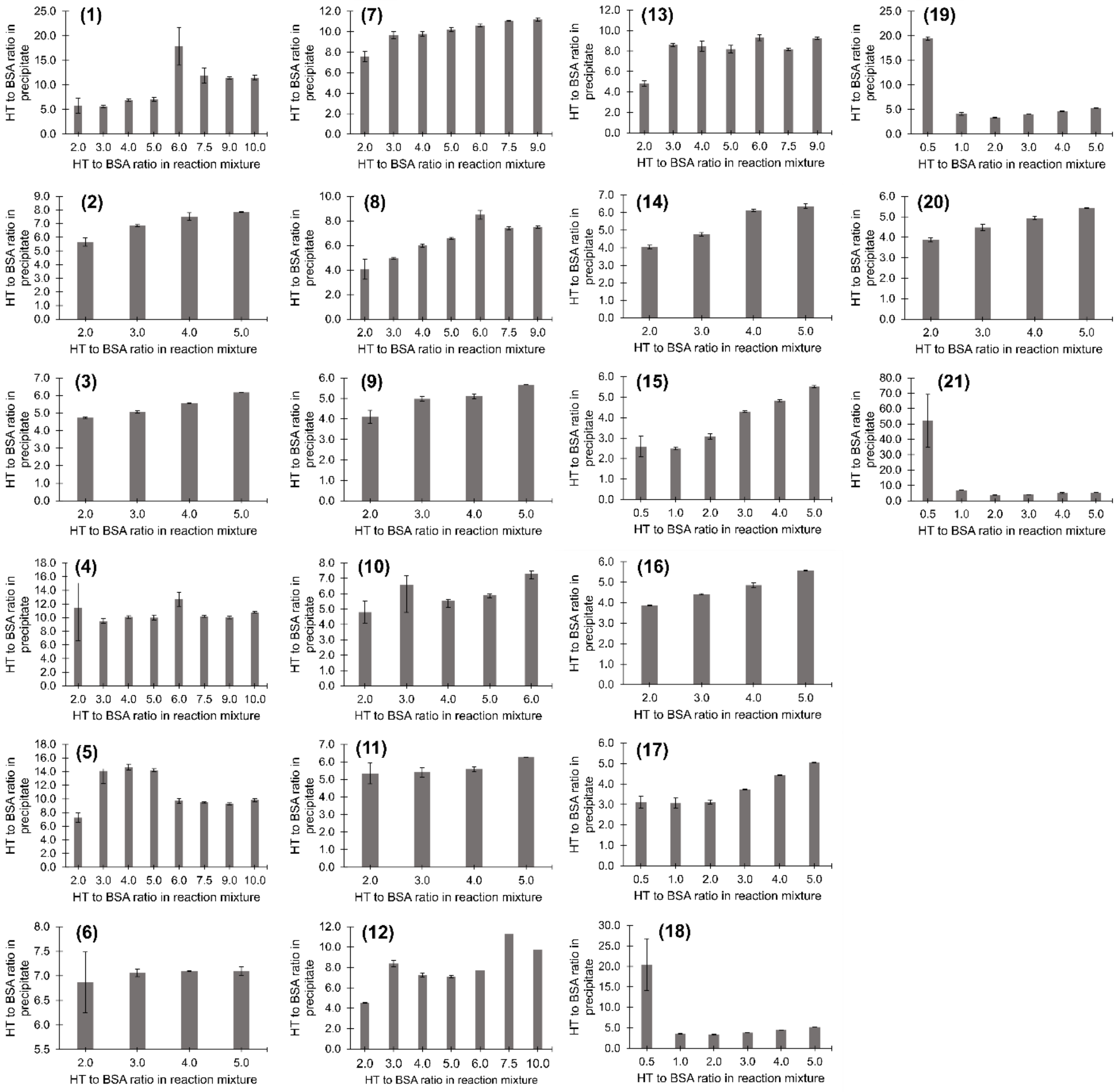

Figure S6. The individual plots of each studied hydrolyzable tannin (HT) showing the initial HT to bovine serum albumin (BSA) ratio versus HT to BSA ratio in the precipitate. (**1**) 1,2,6-tri-*O*-galloyl-β-D-glucose, **(2**) 1,2,3,4,6-penta-*O*-galloyl-β-D-glucose, (**3**) octagalloylglucose, (**4**) tellimagrandin I, (**5**) 1,2-di-*O*-galloyl-4,6-HHDP-β-D-glucose, (**6**) punicalagin, (**7**) geraniin, (**8**) vescalagin, (**9**) castalagin, (**10**) vescavaloninic acid, (**11**) castavaloninic acid, (**12**) stachyurin, (**13**) casuarinin, (**14**) oenothein B, (**15**) oenothein A, (**16**) sanguiin H-6, (**17**) lambertianin C, (**18**) gemin A, (**19**) agrimoniin, (**20**) salicarinin A and (**21**) salicarinin B.
